# Enantioselective Extraction System Containing Binary Chiral Selectors and Chromatographic Enantioseparation Method for Determination of the Absolute Configuration of Enantiomers of Cyclopentolate

**DOI:** 10.1007/s10337-013-2538-z

**Published:** 2013-08-23

**Authors:** Kamila Szwed, Marcin Górecki, Jadwiga Frelek, Monika Asztemborska

**Affiliations:** 1 Institute of Physical Chemistry, Polish Academy of Sciences, Kasprzaka 44/52, 01-224 Warsaw, Poland; 2 Institute of Organic Chemistry, Polish Academy of Sciences, Kasprzaka 44/52, 01-224 Warsaw, Poland

**Keywords:** Column liquid chromatography, Enantioselective extraction, Cyclodextrin, Chiral extraction, Cyclopentolate

## Abstract

The distribution coefficients and enantioseparation of cyclopentolate were studied in an extraction system containing d-tartaric acid ditertbutyl ester in organic phase and 2-hydroxypropyl-β-cyclodextrin (HP-β-CD) in aqueous phase. Various parameters involved in the enantioseparation such as the type and the concentration of chiral selectors, pH value and a wide range of organic solvents were investigated. The maximum enantioselectivity (*α* = 2.13) and optimum distribution coefficients (*K*_R_ = 0.85, *K*_S_ = 0.40) were obtained under the following conditions: 0.10 mol/L HP-β-CD in aqueous phase and 0.20 mol/L d-tartaric acid ditertbutyl ester in decanol as organic phase. Cyclopentolate is present as a racemic mixture to the aqueous phase. The potentially different biological activities of cyclopentolate enantiomers have not been examined yet. Two chiral liquid chromatography methods have been developed for the direct separation of the enantiomers of cyclopentolate. First method was used for the quantification analysis of cyclopentolate enantiomers in aqueous phase. Second method used two chiroptical detectors: electronic circular dichroism (ECD) and optical rotation (OR) for the identification of individual cyclopentolate enantiomers from the organic phase enriched with (*R*)-enantiomer. The absolute stereochemistry was determined by means of the comparison of the experimental and computed ECD spectra and signs of OR. The ECD spectra of chiral analytes were measured *on*-*line* using HPLC-ECD technique.

## Introduction

Separation of enantiomers is a topic of great interest in many branches of science such as pharmaceutical, medicinal chemistry, agrochemicals and food chemistry [1–3]. The fact that enantiomers usually possess different physical and pharmacological properties in biological systems [4] has a strong stimulating influence on the development of new, more efficient enantioseparation methods.

Asymmetric synthesis and chiral resolution are two main methods used for obtaining pure enantiomers. Compared to popular resolution methods, asymmetric synthesis is more expensive and often characterized by low yields [5]. Chiral resolution methods such as chromatographic separation, kinetic resolution, and crystallization resolution also have some disadvantages. Chromatographic methods can separate racemic mixture on an analytical and preparative level, however, they are rather expensive and time-consuming [6]. The main disadvantage of kinetic resolution is the loss of catalytic activity over time [7]. Crystallization is also time-consuming, and the desired compounds are often obtained as derivatives such as diastereomeric salts [8], diastereoisomeric derivatives [9], or inclusion complexes [10]. Chiral extraction is cheaper and easier than other methods. In addition, it can be easily scaled up to industrial scale [11, 12].

Chiral selectors play an important role in the efficiency of enantioseparation.

Tartaric acid derivatives are commonly used as chiral selectors for enantioseparation [13–15]. Nevertheless, these chiral selectors exhibit a limited ability for enantioseparation that is inadequate in context of industrial application. Higher enantioselectivity can be obtained by combination of tartaric acid derivatives with other chiral selectors [16]. Cyclodextrins (CD) are extensively used in separation by virtue of their availability and low cost [17–20]. They are cyclic oligosaccharides that can incorporate molecules of appropriate size into their hydrophobic cavity, forming the inclusion complexes.

Several papers on the enantioselective extraction system containing tartaric acid derivatives and CD were also studied [21–23].

**Fig. 1:**
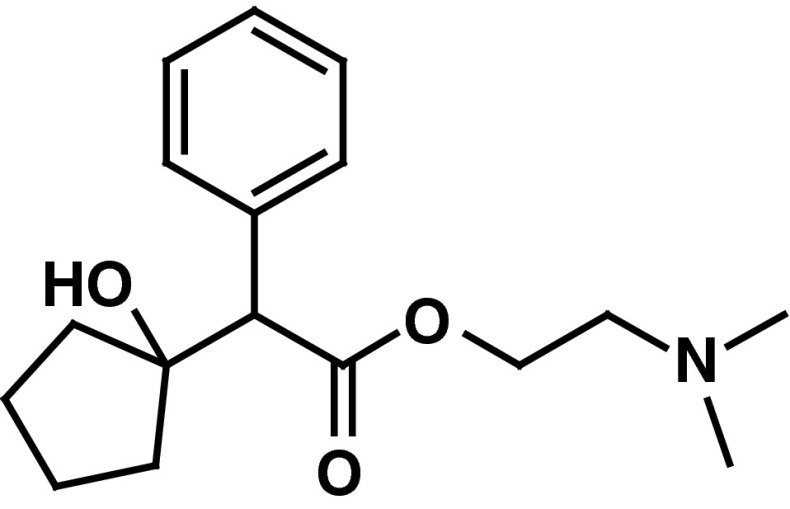
Chemical structure of cyclopentolate

The objective of this study was to investigate tartaric acid derivatives and β-cyclodextrin derivatives as binary chiral selectors in extraction of racemic cyclopentolate, which, as a mydriatic agent, is commonly used during pediatric eye examinations [24] (Fig. 1). Cyclopentolate is added as racemic mixture to the aqua phase. Various parameters involved in enantioseparation of cyclopentolate were investigated, such as type and concentration of chiral selectors, pH values, type of organic solvent.

## Materials and Methods

### Materials

β-Cyclodextrin (β-CD) and trimethyl-β-cyclodextrin (TM-β-CD) were supplied by Chinoin (Budapest, Hungary). Hydroxypropyl-β-cyclodextrin (HP-β-CD), dimethyl-β-cyclodextrin (DM-β-CD) and cyclopentolate (cyclopentolate) were all obtained from Aldrich Co. (St. Louis, MO, USA). Sodium 1-octanesulfonate, l-tartaric acid ditertbutyl ester, d- and l-tartaric acid diethyl ester were bought from TCI Europe (Zwijndrecht, Belgium). d-tartaric acid ditertbutyl ester was purchased from Santa Cruz Biotechnology (Santa Cruz, CA, USA). d-, l-tartaric acid diisobutyl ester and d-, l-tartaric acid dibutyl ester were synthesised as described in literature [25].

### Analytical Methods

The quantification analysis of cyclopentolate enantiomers in aqueous phase was performed by HPLC using a Waters (Milford, MA, USA) Model 515 pump, 717 plus autosampler with 1 μL loop and a Waters UV/VIS detector Model 2,487 (detection: 220 nm). The mobile phase was prepared by dissolving 15 mM of β-CD, 1 mM sodium 1-octanesulfonate, 10 mM NaH_2_PO_4_ in aqueous ethanolic solutions (20 % (*v/v*) EtOH-water) and finally adjusted to pH 2.0 by addition of phosphoric acid. The column was Luna 5 μm C18 (2) 100 A 150 mm × 1 mm (Phenomenex, Torrance, CA, USA). Flow rate of mobile phase was 0.04 mL/min. Chromatographic measurements were performed at the 20 °C.

The identification of cyclopentolate enantiomers in organic phase was performed using a Jasco analytical HPLC system equipped with PU-2089 Plus pump with inner degasser, column oven, a 20 μL sample loop, and MD-2010 high resolution diode array UV–VIS detector (600–195 nm). Chiroptical detections were performed using ECD and OR detectors. An ECD chromatogram was obtained at a fixed wavelength by setting the flow cell attachment in the sample chamber of ECD Jasco J-815 spectrometer, and then introducing an eluant from HPLC. The optical rotation chromatogram was recorded using the Jasco OR-2090 detector equipped with 150 W Hg–Xe-lamp working in the range of white light (900–350 nm). The signal was processed by ChromNAV Jasco software. The column was Chiralpak AD (Daicel, Tokyo, Japan) column (250 mm × 4.6 mm, 5 μm). The mobile phase was ^*i*^PrOH:hexane (90:10, *v/v*) with a flow rate of 1 mL/min. The column temperature was set at 20 °C.

The *on*-*line* electronic circular dichroism (ECD) spectra were recorded between 350 and 200 nm at room temperature. Solutions at maximum ECD absorption were trapped in the flow cell attachment fixed to the sample chamber of ECD Jasco J-815 spectrometer. All spectra were recorded using 100 nm/min scanning speed, a step size of 0.2 nm, a bandwidth of 2 nm, a response time of 0.5 s, and an accumulation of five scans. The spectra were background corrected using mobile phase.

### Extraction Experiments

The aqueous phase contained 0.05 mM/L of racemic cyclopentolate and various concentrations of β-CD derivatives (HP-β-CD, DM-β-CD or TM-β-CD) in 0.10 mol/L Na_2_HPO_4_/H_3_PO_4_ buffer solution. d- and l-tartaric acid derivatives were used as extractants and dissolved in the organic phase. 5 mL of organic and 5 mL of aqueous phases were placed in 25 mL glass-stoppered tube and shaken sufficiently (10 h) at a constant temperature (20 °C) to reach equilibrium. After the phase separation, the concentration of cyclopentolate enantiomers in the aqueous phase was determined by HPLC. The distribution coefficients of (*S*)- and (*R*)-forms of cyclopentolate between the organic and aqueous phases (*K*_S_, *K*_R_) and enantioselectivity (*α*) were evaluated according to the following equations:

1 $$ K_{\text{R}} = \frac{{{\text{Concentration of }}(R) - {\text{cyclopentolate in organic phase}}}}{{{\text{Concentration of }}(R) - {\text{cyclopentolate in aqueous phase}}}} $$

2 $$ K_{\text{S}} = \frac{{{\text{Concentration of }}\left( S \right) - {\text{cyclopentolate in organic phase}}}}{{   {\text{Concentration of }}\left( S \right) - {\text{cyclopentolate in aqueous phase}}}} $$

3 $$ \alpha = \frac{{K_{\text{R}} }}{{K_{\text{S}} }} $$

### Computational Details

The conformational search was done using ComputeVOA [26] program with Merck Molecular Force Field 94 (MMF94) within 5 kcal/mol energy windows, and then all structures (143) were submitted to the Gaussian 09 program [27] for a single-point energy calculation at B3LYP/6-31G(d) DFT level of theory. In order to improve confidence level for conformers found (17) second optimization was performed at B3LYP/aug-cc-pVDZ level in the 3 kcal/mol energy window. Then, finally these 17 structures were used for simulation of UV/ECD spectra using B3LYP/aug-cc-pVDZ level. Rotatory strengths were calculated using both length and velocity representations. The differences between the length and velocity of calculated values of rotatory strengths were <5 %, and for this reason, only the velocity representations (*R*_vel_) were taken into account. The ECD spectra were simulated by overlapping Gaussian functions for each transition by means of the SpecDis program [28]. The final spectra were Boltzmann averaged (*T* = 293 K) according to the population percentages of individual conformers based on the relative SCF energies. The calculated UV spectra were red-shifted by 18 nm in relation to the experimental, and consequently ECD spectra were also wavelength corrected. A Gaussian band-shape was applied with 0.48 eV as a half-height width. Very similar results were obtained using B3LYP/6-311++G(2d,2p) and CAM-B3LYP/aug-cc-pVDZ level of theory.

In order to predict OR sign calculations were made for the same group of 17 conformers as ECD, in Gaussian 09 package at 589 nm wavelength using the B3LYP/aug-cc-pVDZ level.

## Results and Discussion

### The Method for Identification of Absolute Configuration of Enantiomers of Cyclopentolate

**Fig. 2:**
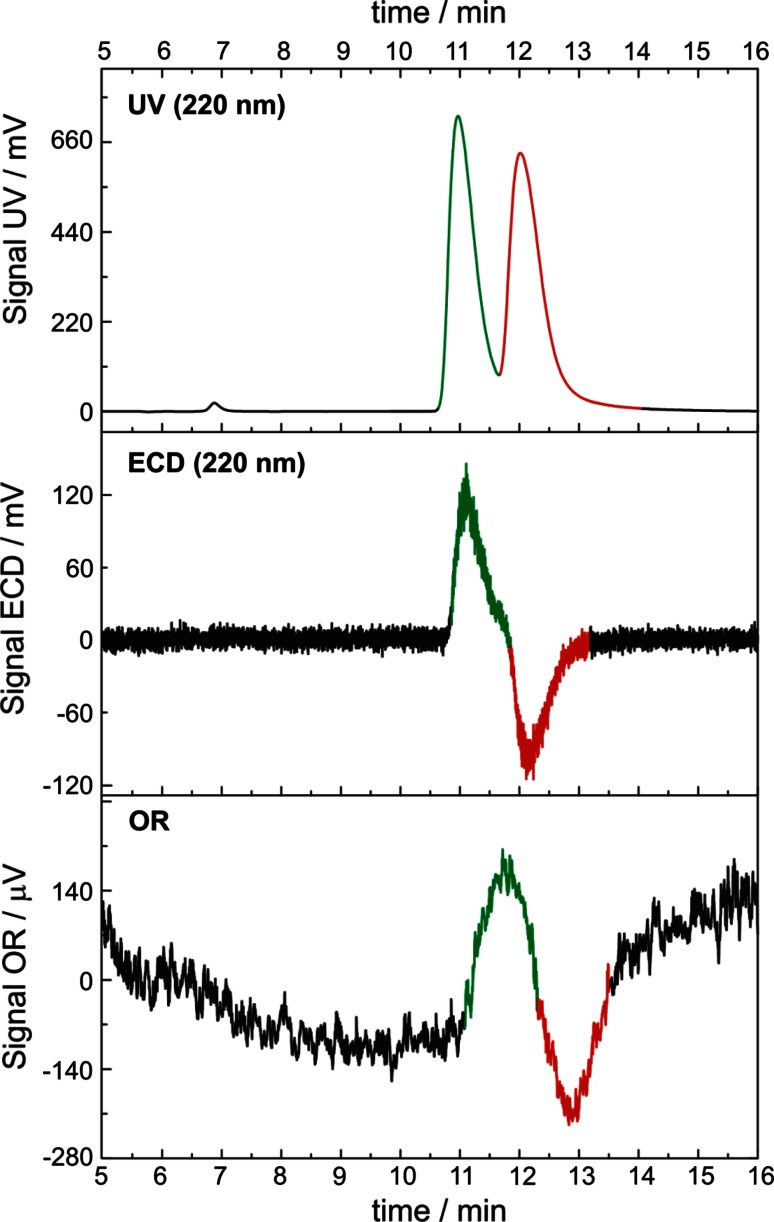
UV (*upper*) and ECD (*middle*) and OR (*lower*) chromatograms of racemic cyclopentolate on the AD Chiralpak column. Chromatographic conditions are described in experimental section

The enantioselective separation of racemic cyclopentolate was successfully performed using Chiralpak AD column by application of two chiral detectors: ECD and OR. The first eluted enantiomer displays a positive ECD and OR values while second one exhibits opposite signs for both chiroptical methods (Fig. 2).

**Fig. 3:**
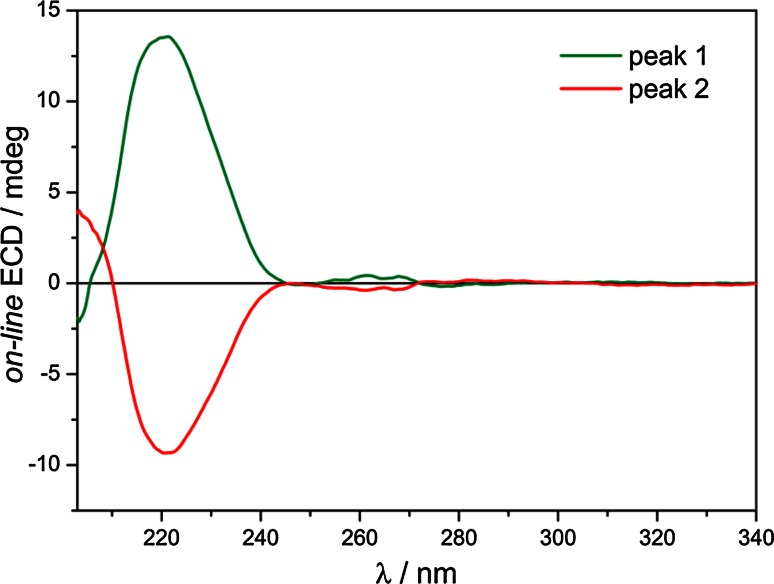
ECD spectra measured *on*-*line* of two elution peaks at 10.9 and 12.0 min, respectively

The ECD spectra measured *on*-*line* of two elution peaks are shown in the Fig. 3. The first eluted enantiomer of cyclopentolate displays two positive ECD bands: a very weak one at 262 nm and a strong one at 221 nm. The first band at 262 nm with well-defined vibrational fine structure is related to the ^1^ *L*_b_ (π → π*) transition which involves orbitals of chirally perturbed benzene ring. The second one at 221 nm is an admixture of the ^1^ *L*_a_ (π → π*) transition from aromatic ring and of the ester *n* → π* transition.

The absolute configuration (AC) of eluted enantiomers were determined on the basis of the comparison of the experimental and computed ECD spectrum and sign of OR. The calculations were performed using the Time Dependent Density Functional Theory (TDDFT) method for an arbitrarily chosen (*R*)-enantiomer. This combined analysis has already proven efficient and reliable for the assignment of the AC of various chiral organic molecules [29–31].

**Fig. 4:**
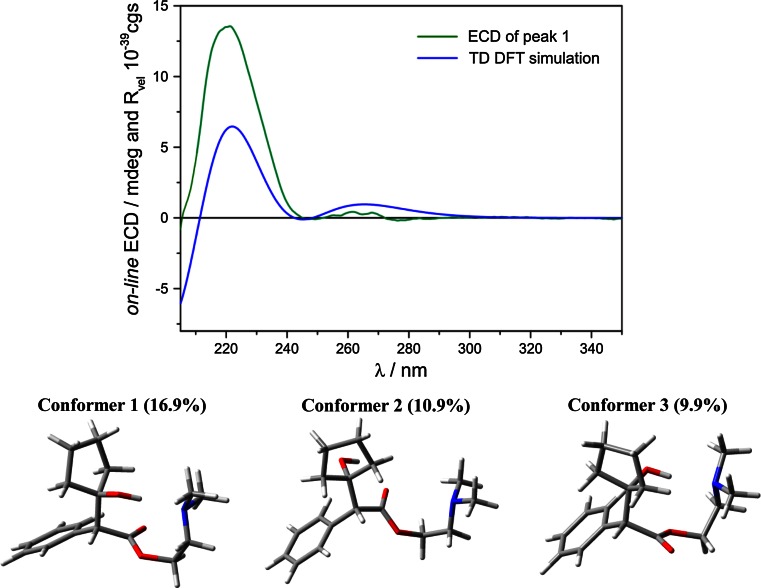
*Top* Computed ECD spectrum at the B3LYP/aug-cc-pVDZ level of theory obtained as a population-weighted sum at 293 K of individual conformers of (*R*)-cyclopentolate compared to measured *on*-*line* ECD spectrum of the first peak eluted at 10.9 min. *Bottom* Optimized structures of the three lowest-energy conformers

In order to assure a reliable AC assignment, first a thorough conformational analysis by molecular mechanics (MMF94 force field) was carried out to find the lowest-energy conformers. Then, all of the conformers were optimized at B3LYP/6-31G(d) level of theory. In order to refine the data, the reoptimization was performed at a higher level (B3LYP/aug-cc-pVDZ). Finally, 17~ conformers within the range of 3 kcal/mol were selected for ECD and OR calculations. The OR calculations were carried out at the wavelength of 589 nm. As can be seen in Fig. 4, the Boltzmann averaged ECD spectrum of the (*R*)-enantiomer is in an excellent agreement with the experimental spectrum of the first eluted peak.

An additional proof of this AC assignment comes from calculated value of OR at 589 nm. For the same set of conformers, as for ECD spectra, the simulated Boltzmann average value of OR is positive.

In conclusion, this combined experimental and theoretical analysis of ECD and OR data allowed us to confidently assign the AC to be (*R*) for the first eluted peak and (*S*) for the second one in accordance with experimental data. This method was used only to determine which enantiomer is enriched in the organic phase. It was found that in the organic phase (*R*)-cyclopentolate was present in excess.

### Effect of Organic Solvents on Chiral Extraction

Table 1 shows the influence of various organic solvents on distribution coefficients and enantioselectivity. The aqueous phase contains 0.05 mmol/L racemic cyclopentolate, 0.10 mol/L Na_2_HPO_4_/H_3_PO_4_ buffer and 0.10 mol/L HP-β-CD. There is no extractant in organic phase. The large influence of organic solvent on the extraction efficiency has also been investigated in previous works [32, 33].

**Table 1:** Effect of organic solvent on *K* and *α*

Organic solvent	*K*_R_	SD	*K*_S_	SD	*α*	SD
1,2-Dichloroethane	0.050	0.037	0.041	0.035	1.220	0.053
Dichlorobenzen	0.035	0.022	0.020	0.025	1.750	0.076
Cyclohexane	0.087	0.020	0.011	0.018	7.909	0.118
*n*-Hexanol	0.365	0.058	0.258	0.051	1.411	0.064
*n*-Heptanol	0.396	0.054	0.272	0.053	1.465	0.091
*n*-Octanol	0.461	0.060	0.264	0.065	1.745	0.097
*n*-Decanol	0.487	0.063	0.276	0.059	1.831	0.086

Aqueous phase: [HP-β-CD] = 0.10 mol/L, [cyclopentolate] = 0.05 mM/L, pH 6.0

Table 1 illustrates, extraction performance of different kinds of organic solvents. When 1,2-dichloroethane, dichlorobenzene and cyclohexane were used as solvent, very low distribution coefficients were obtained. This may be caused by the fact that the cyclopentolate is poorly extracted into the organic phase.

When alcohol groups were used as solvents, relatively higher distribution coefficients were obtained. The distribution coefficients and enantioselectivity increase with the elongation of length of alkyl chain of alcohol.

The organic solvents can penetrate into the aqueous phase and can form complexes of different stabilities with β-HP-CD [34]. In addition, the more hydrophilic solvents more easily penetrate into aqueous phase. The organic solvent in the aqueous phase can cause a displacement of the analyte from the CD cavity. The result is a decrease in enantioselectivity.

When cyclohexane was used, high enantioselectivity was obtained but low distribution coefficients were found. Distribution coefficients for cyclopentolate enantiomers were higher with alcohol groups. According to these observations, decanol was chosen as a suitable organic solvent giving the highest distribution coefficients with satisfied enantioselectivity.

### Effect of β-Cyclodextrin Derivatives

The CD are capable of forming inclusion complexes with compounds having a size, shape and polarity compatible with the dimensions of their cavity. Cyclopentolate can form inclusion complexes with the following derivatives of β-CD: HP-β-CD, DM-β-CD, TM-β-CD. Stability constants of complexes β-CD derivatives (HP-β-CD, DM-β-CD, TM-β-CD) with cyclopentolate were determined by capillary electrophoresis technique [35] Cyclopentolate forms the most stable complexes with DM-β-CD (1,769.2 L/mol) and the least stable with the TM-β-CD (7.93 L/mol). Stability constant complex of cyclopentolate with HP-β-CD (590.1 L/mol) has a value in between.

The distribution coefficients and enantioselectivity were determined by chiral extraction (0.10 mol/L β-CD derivatives in aqueous phase and no addition of tartaric acid derivatives to organic phase).

In Table 2, the highest enantioselectivity and distribution coefficients of (*R*)-enantiomer were achieved when HP-β-CD was used, but with lowest distribution coefficients of (*S*)-enantiomer. However, when DM-β-CD and TM-β-CD were used significantly lower enantioseparation was achieved.

**Table 2:** Effect of β-CD derivatives

β-CD derivatives	*K*_R_	SD	*K*_S_	SD	*α*	SD
HP-β-CD	0.52	0.074	0.27	0.051	1.96	0.131
DM-β-CD	0.48	0.090	0.35	0.076	1.33	0.068
TM-β-CD	0.45	0.076	0.30	0.050	1.49	0.095

Aqueous phase: [β-CD derivatives] = 0.10 mol/L, [cyclopentolate] = 0.05 mmol/L, pH 6.0

It can also be found in Table 2 that *K*_R_ are always higher than *K*_S_, which indicates that HP-β-CD preferentially interacts with (*S*)-cyclopentolate and this complex is retained in the aqueous phase.

Among the three β-CD derivatives, HP-β-CD has the highest enantioselectivity and was chosen as a chiral selector in aqueous phase. It was reported that HP-β-CD was also previously used for enantioselective extraction, among others with binary selector systems [22, 36].

### Effect of Tartaric Acid Derivatives

The distribution coefficients and enantioselectivity were also determined in chiral extraction containing 0.10 mol/L HP-β-CD in aqueous phase and 0.20 mol/L-tartaric acid derivatives in organic phase.

Table 3 shows that the distribution coefficients and enantioselectivity increased when tartaric acid derivatives were added to the organic phase. In this case the enantioselective extraction system has stronger chiral separation ability than the monophasic extraction.

**Table 3:** Effect of tartaric acid derivatives

Tartaric acid derivatives	*K*_R_	SD	*K*_S_	SD	*α*	SD
l-Tartaric acid diethyl ester	0.67	0.064	0.34	0.068	1.97	0.043
d-Tartaric acid diethyl ester	0.75	0.079	0.38	0.070	1.98	0.056
l-Tartaric acid dibutyl ester	0.66	0.120	0.34	0.108	1.94	0.099
d-Tartaric acid dibutyl ester	0.68	0.118	0.35	0.087	1.95	0.127
l-Tartaric acid diisobutyl ester	0.82	0.096	0.40	0.093	2.02	0.069
d-Tartaric acid diisobutyl ester	0.85	0.073	0.42	0.062	2.06	0.091
l-Tartaric acid ditertbutyl ester	0.82	0.086	0.39	0.071	2.09	0.068
d-Tartaric acid ditertbutyl ester	0.85	0.098	0.40	0.065	2.13	0.082

Organic phase: (tartaric acid derivatives) = 0.2 mol/L, aqueous phase: [HP-β-CD] = 0.10 mol/L, [cyclopentolate] = 0.05 mmol/L, pH 6.0

The use of d-tartaric acid derivatives as an additive led to the higher *K*_R_ value than *K*_S_ value observed when the l-tartaric acid derivatives were used. This result indicates that the d-tartraic acid derivatives have stronger recognition ability for (*R*)-cyclopentolate than for (*S*)-cyclopentolate.

Chiral extraction performance is related to the structure of chiral selector. It is very important to investigate the influence of different branched alkyl side chains of tartaric acid derivatives on the distribution coefficients and enantioselectivity. The distribution coefficients and enantioselectivity increase slightly with the degree of alkyl chain branching. Because the bulky alcohols might allow for a more stereospecific and stronger interaction with cyclopentolate, which increases the stabilization of complexes of chiral selector with cyclopentolate molecules. Consistent with observations by Prelog et al. [37] the highest enantioselectivity is provided by extensive tartaric acid derivatives. However, the differences in the values of the distribution coefficients and enantioselectivity are too small for any discussion structural effects on the chiral recognition mechanism. Therefore, d-tartaric acid ditertbutyl ester was chosen as the chiral selector in the organic phase.

### Effect of Chiral Selector Concentrations

**Fig. 5:**
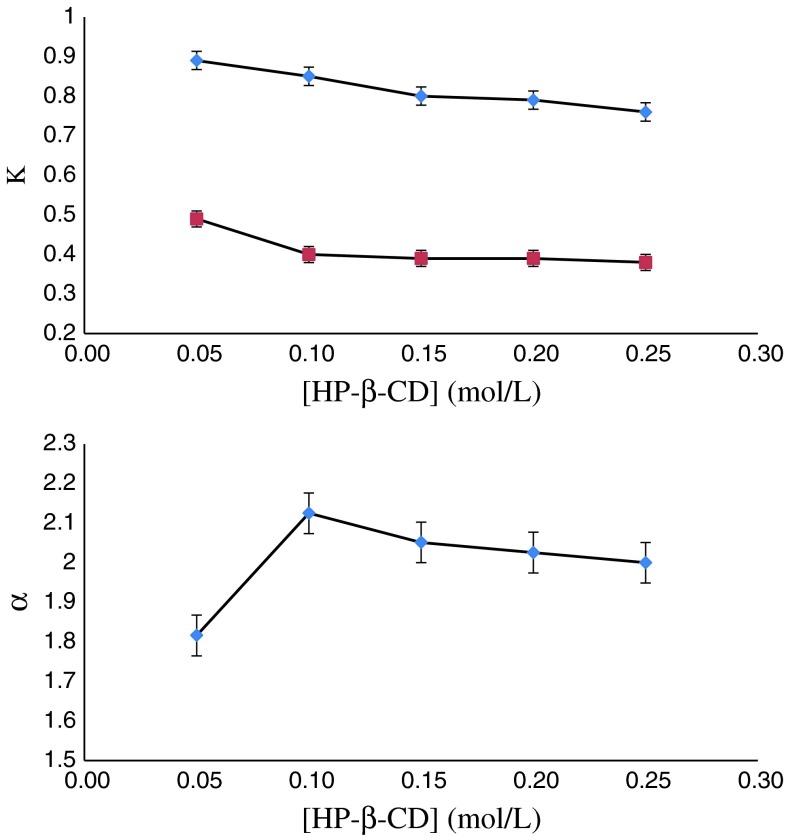
Effect of HP-β-CD concentration on *K* and *α*. Organic phase: [d-tartaric acid ditertbutyl ester] = 0.20 mol/L

**Fig. 6:**
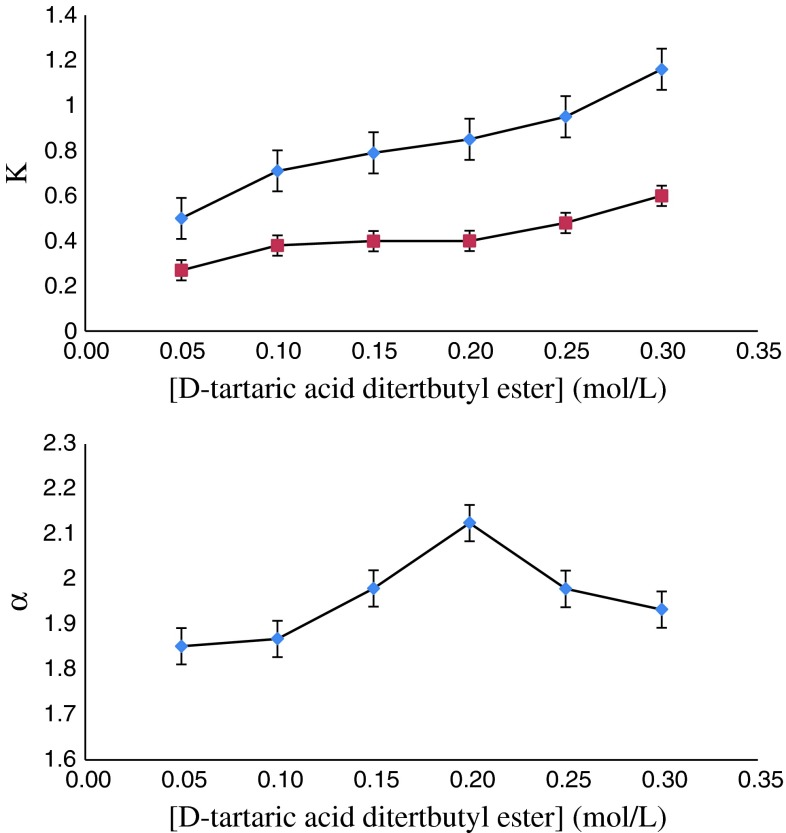
Effect of d-tartaric acid ditertbutyl ester concentration on *K* and *α*. Aqueous phase: [HP-β-CD] = 0.10 mol/L, [cyclopentolate] = 0.05 mM/L, pH 6.0

Figures 5 and 6 both summarize the effect of concentration of HP-β-CD in the aqueous phase and d-tartaric acid ditertbutyl ester in the organic phase on the distribution coefficients and enantioselectivity. With the increase of HP-β-CD concentration, the distribution coefficients for cyclopentolate enantiomers decrease, which can be explained by the higher amount of complexes formed in the aqueous phase. The enantioselectivity increases steadily, up to the concentration of HP-β-CD at 0.10 mol/L. When the concentration of HP-β-CD is over 0.10 mol/L the enantioselectivity decreases subtly. The behavior of enantioselectivity can be explained by the results of the cooperation of HP-β-CD in the aqueous phase and d-tartaric acid ditertbutyl ester in the organic phase, which is in accordance with below results.

On the contrary, with increase of d-tartaric acid ditertbutyl ester concentration, the distribution coefficients for cyclopentolate enantiomers increase and the enantioselectivity increases up to the concentration of d-tartaric acid ditertbutyl ester at 0.20 mol/L. When the concentration of d-tartaric acid ditertbutyl ester exceeds 0.20 mol/L, the enantioselectivity begins to decrease.

This is because a large amount of complexes for cyclopentolate enantiomers were formed in the organic phase which led to an increase of the distribution coefficients and the enantioselectivities were the results of the cooperation of HP-β-CD in the aqueous phase and d-tartaric acid ditertbutyl ester in the organic phase.

As seen in Figs. 2 and 3, the enantioselectivity reaches a maximum at the ratio 2:1 in the molar concentration of d-tartaric acid ditertbutyl ester to HP-β-CD. A possible explanation of this behavior is that HP-β-CD can form inclusion complexes with cyclopentolate enantiomers, and the complexation ability of HP-β-CD toward (*S*)-cyclopentolate is higher than toward (*R*)-cyclopentolate. This statement is backed by experimental data presented in Table 2 (*K*_R_ = 0.52, *K*_S_ = 0.27).

### Effect of pH

**Fig. 7:**
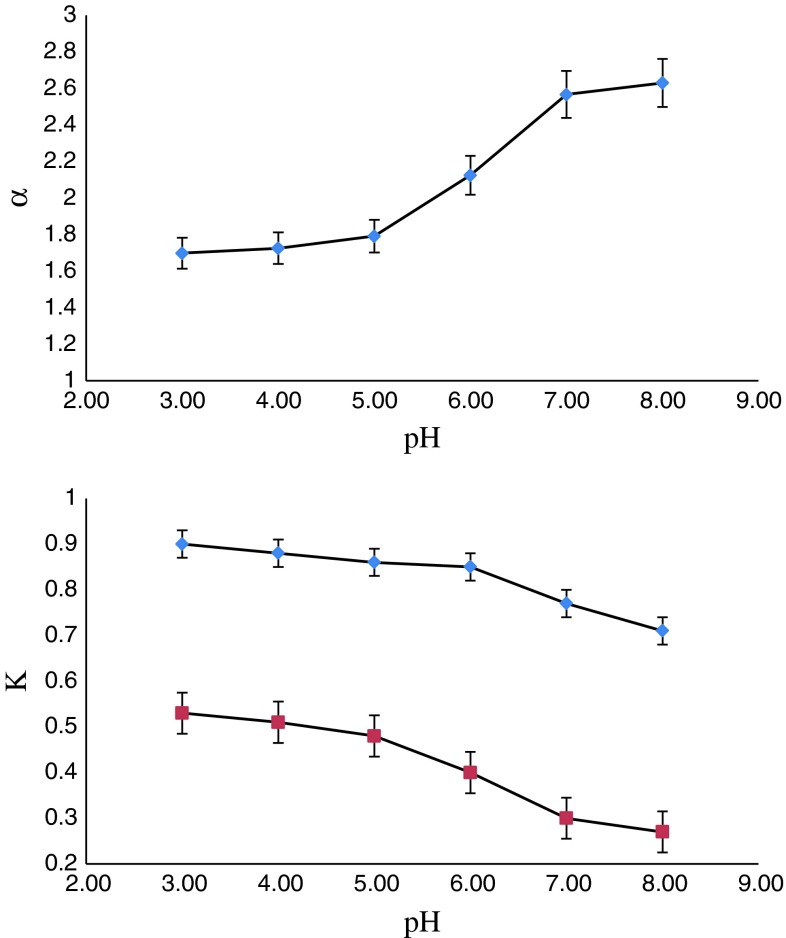
Effect of pH on K and *α*. Aqueous phase: [HP-β-CD] = 0.10 mol/L, [cyclopentolate] = 0.05 mM/L. Organic phase: [d-tartaric acid ditertbutyl ester] = 0.20 mol/L

Figure 7 shows the effect of pH on the distribution coefficients and enantioselectivity of cyclopentolate enantiomers. As seen in Fig. 2, distribution coefficients decrease with increase of pH. On the other hand, the enantioselectivity increases with increase of pH.

The possible reasons may be that the ratio between protonated and unprotonated cyclopentolate decreases with the rise of pH value. d-tartaric acid ditertbutyl ester has the chiral recognition ability limited mainly for the neutral molecule of cyclopentolate. Ionic cyclopentolate only exists in aqueous phase. In search for the optimum combination of the highest distribution coefficients and favorable enantioselectivity, the pH 6.0 was an appropriate choice for extraction of cyclopentolate enantiomers.

## Conclusions

Liquid–liquid extraction proved to be a powerful tool to achieve chiral separation.

In order to obtain sufficient enantioselectivity, it is necessary to optimize conditions for the chiral extraction. The type and concentration of chiral selectors, the pH value, the choice of organic solvent were clearly identified as important factors influencing enantioselectivity.

**Fig. 8:**
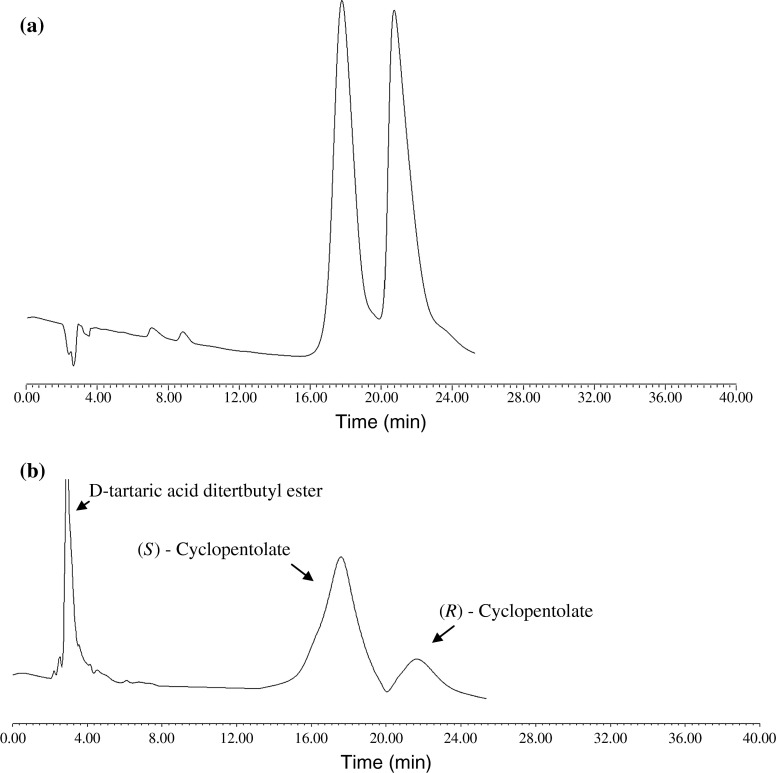
Chromatograms of cyclopentolate enantiomers; **a** before enantioselective extraction **b** after enantioselective extraction under optimal conditions (aqueous phase: [HP-β-CD] = 0.10 mol/L, [cyclopentolate] = 0.05 mM/L, pH 6.0. Organic phase (decanol): [d-tartaric acid ditertbutyl ester] = 0.20 mol/L)

As seen in Fig. 8, high enantioselectivity and distribution coefficients for cyclopentolate were obtained using 0.10 mol/L HP-β-CD and 0.20 mol/L d-tartaric acid ditertbutyl ester in decanol as organic phase. Optimum pH was about six for the chiral separation of cyclopentolate. The enantiomeric excess is 61.13 % for the aqueous phase under the optimal condition.

## References

[1] Maier NM, Franco P, Lindner WJ (2001). J Chromatogr A.

[2] Afonso CA, Crespo JG (2004). Angew Chem Int Ed.

[3] Breuer M, Ditrich K, Habicher T, Hauer B, Kesseler M, Sturmer R, Zelinski T (2004). Angew Chem Int Ed.

[4] Lacour J, Goujon-Giglinger C, Torche-Haldimann S, Jodry J (2000). Angew Chem Int Ed.

[5] Kotha S (1994). Tetrahedron.

[6] Schurig V (2001). Separation of enantiomers by gas chromatography. J Chromatogr A.

[7] Vedejs E, Jure M (2005). Angew Chem Int Ed.

[8] Liao J, Peng XH, Zhang JH, Yu KB, Cui X, Zhu J, Deng JG (2003). Org Biomol Chem.

[9] Alexakis A, Frutos JC, Mutti S, Mangeney P (1994). J Org Chem.

[10] Deng JG, Chi YX, Fu FM, Cui X, Yu KB, Zhu J, Jiang YZ (2000). Tetrahedron: Asymmetry.

[11] Steensma M, Kuipersa NJM, Haan AB, Kwant G (2007). Chem Eng Sci.

[12] Koska J, Haynes CA (2001). Chem Eng Sci.

[13] Heldin E, Lindner K, Pettersson C, Lindner W, Rao R (1991). Chromatogr.

[14] Goswami S, Jana S, Dey S, Razak IA, Fun H (2006). Supramol Chem.

[15] Huang K, Jiao F, Liu S (2006). Lat Am Appl Res.

[16] Jiao FP, Chen XQ, Hu WG (2007). Chem Pap-Chem Zvesti.

[17] Armstrong DW, Jin HL (1987). Anal Chem.

[18] Jiao FP, Huang KL, Ning FR (2006). Sep Sci Technol.

[19] Ferreira Q, Coelhoso IM, Ramalhete N (2006). Sep Sci Technol.

[20] Chetana P, Philip JM, Peter DC (1998). J Chromatogr A.

[21] Jiao F, Chen X, Jiang X (2009). Iran J Chem Chem Eng.

[22] Tang K, Song L, Liu Y, Miao J (2012). J Chem Eng.

[23] Tang K, Song L, Liu Y, Pan Y, Jiang X (2010). J Chem Eng.

[24] Ismail EE, Rouse MW, Land PN (1994). Optom Vis Sci.

[25] Heldin E, Lindner KJ, Pettersson C, Lindner W, Rao R (1991). Chromatographia.

[26] ComputeVOA™ comprehensive software, BioTools, Inc, Jupiter, FL, USA

[27] Gaussian 09, Revision A02, Frisch MJ Trucks GW, Schlegel HB, Scuseria GE, Robb MA, Cheeseman JR, Scalmani G, Barone V, Mennucci B, Petersson GA, Nakatsuji H, Caricato M, Li X, Hratchian, HP, Izmaylov, AF, Bloino, J, Zheng, G, Sonnenberg, J L, Hada, M, Ehara, M, Toyota, K, Fukuda, R, Hasegawa, J, Ishida, M, Nakajima, T, Honda, Y, Kitao, O, Nakai, H, Vreven, T, Montgomery Jr, JA, Peralta, JE, Ogliaro, F, Bearpark, M, Heyd, JJ, Brothers, E, Kudin, KN, Staroverov, VN, Kobayashi, R, Normand, J, Raghavachari, K, Rendell, A, Burant, JC, Iyengar, SS, Tomasi, J, Cossi, M, Rega, N, Millam, JM, Klene, M, Knox, JE, Cross, JB, Bakken, V, Adamo, C, Jaramillo, J, Gomperts, R, Stratmann, RE, Yazyev, O, Austin, AJ, Cammi, R, Pomelli, C, Ochterski, JW, Martin, RL, Morokuma, K, Zakrzewski, VG, Voth, GA, Salvador, P, Dannenberg, JJ, Dapprich, S, Daniels, AD, Farkas, O, Foresman, JB, Ortiz, JV, Cioslowski, J, Fox DJ, (2009) Gaussian, Inc, Wallingford CT

[28] Specdis v. 1.53, Bruhn T, Hemberger Y, Schaumlöffel A, Bringmann G (2012) University of Würzburg, Germany

[29] Di Bari L, Pescitelli G, Salvadori P, Rovini M, Anzini M, Cappelli A, Vomero S (2006). Tetrahedron: Asymmetry.

[30] Polavarapu PL, Frelek J, Woźnica M (2011). Tetrahedron: Asymmetry.

[31] Kołodziejska R, Górecki M, Frelek J, Dramiński M (2012). Tetrahedron: Asymmetry.

[32] Steensme M, Kuipers N, Haan AB, Kwant G (2006). J Chem Technol Biotechnol.

[33] Tang K, Yi J, Liu Y, Jiang X, Pan Y (2009). Chem Eng Sci.

[34] Másson M, Karlsson FJ, Valdimarsdóttir M, Magnúsdóttir K, Loftsson T (2007). J Incl Phenom Macrocycl Chem.

[35] Kwaterczak A, Duszczyk K, Bielejewska A (2009). Anal Chim Acta.

[36] Sunsandeea N, Pancharoena U, Rashatasakhonb P, Ramakulc P, Leepipatpiboond N (2013). Separ Sci Technol doi.

[37] Prelog V, Mutak S, Kovacevic K (1983). Chim Acta.

